# Results of a study on the correlation between students’ motivation to achieve success and anxiety

**DOI:** 10.1192/j.eurpsy.2026.11227

**Published:** 2026-07-31

**Authors:** P. Sarantuya, M. Nyamaa, B. Yuri, B. Dolgorsuren, T. Urtnasan

**Affiliations:** 1 Graduate School of Education, Graduate University of Mongolia; 2 Medical Department, School of Medicine, Etugen University; 3 Health center, Mongolian National University of Education, Ulaanbaatar, Mongolia

## Abstract

**Introduction:**

From an educational perspective, motivation is a multifaceted construct that is closely related to learning and academic success. In other words, academic success motivation is a student’s tendency to act in a certain way and evaluate themselves. Additionally, this study was conducted considering the problems faced by the education system, including poor quality of education, which leads to factors such as pessimism, anxiety, and depression that reduce students’ motivation to learn and increase the risk of reduced success motivation.

**Objectives:**

To investigate the motivation for success in nursing students in relation to anxiety.

**Methods:**

The study employed a descriptive research design. The study was conducted using a random sampling formula from all undergraduate nursing students, including n=160 students, and included 16 questions from the standard general section developed by researcher Yu.M. Orlov, 22 questions to assess motivation to achieve, and 40 questions to determine anxiety developed by Spielberga Khanin. The survey was conducted in 30 minutes at number 101, 4th place, from March 12 to March 20.

**Results:**

36.9% had a low level of motivation to achieve success, 43.8% had an average level of motivation to achieve success, and 19.4% had a high level of motivation to achieve success. 9.4% had a high level of anxiety, 19.4% had a moderate level of anxiety, and 71.3% had a low level of anxiety, respectively. 10% of the first-year students had a high level of motivation to achieve success, 56.25% had an average level of motivation to achieve success, and 33.75% had a low level of motivation to achieve success. 28.75% of the 4th-year students had a high level of motivation to succeed, 31.25% had an average level of motivation to succeed, and 40% had a low level of motivation to succeed. 5% of the first-year students had high anxiety, 18.75% had moderate anxiety, and 76.25% had low anxiety, while 13.75% of the fourth-year students had high anxiety, 20% had moderate anxiety, and 66.25% had low anxiety. There was a weak positive correlation between the achievement motivation score and anxiety score (r=0.28, p=0.00) and a statistically significant relationship. In simple linear regression analysis, an increase in the achievement motivation score by one affected the anxiety score by 42%.

**Conclusions:**

The students who participated in the study have low desire and ambition to achieve success, and there are no students who are not anxious. Motivation to achieve success and anxiety are high in the fourth grade. Motivation to achieve success and anxiety are related, and motivation affects anxiety.

**Disclosure of Interest:**

None Declared

